# Expression Patterns Divergence of Reciprocal F_1_ Hybrids Between *Gossypium hirsutum* and *Gossypium barbadense* Reveals Overdominance Mediating Interspecific Biomass Heterosis

**DOI:** 10.3389/fpls.2022.892805

**Published:** 2022-07-01

**Authors:** Tengyu Li, Fuqiu Wang, Muhammad Yasir, Kui Li, Yuan Qin, Jing Zheng, Kun Luo, Shouhong Zhu, Hua Zhang, Yurong Jiang, Yongshan Zhang, Junkang Rong

**Affiliations:** ^1^The Key Laboratory for Quality Improvement of Agricultural Products of Zhejiang Province, Zhejiang Agriculture and Forestry University, Hangzhou, China; ^2^State Key Laboratory of Cotton Biology, Institute of Cotton Research, Chinese Academy of Agricultural Sciences, Anyang, China; ^3^National Key Laboratory of Crop Genetic Improvement, Huazhong Agricultural University, Wuhan, China; ^4^Institute of Food and Nutrition Development, Ministry of Agriculture and Rural Affairs, Chinese Academy of Agricultural Sciences, Beijing, China

**Keywords:** cotton, interspecific hybrid, biomass vigor, transcriptome analysis, overdominant

## Abstract

Hybrid breeding has provided an impetus to the process and achievement of a higher yield and quality of crops. Interspecific hybridization is critical for resolving parental genetic diversity bottleneck problems. The reciprocal interspecific hybrids and their parents (*Gossypium hirsutum* and *Gossypium barbadense*) have been applied in this study to elucidate the transcription regulatory mechanism of early biomass heterosis. Phenotypically, the seed biomass, plant height over parent heterosis, leaf area over parent heterosis, and fresh and dry biomass were found to be significantly higher in hybrids than in parents. Analysis of leaf areas revealed that the one-leaf stage exhibits the most significant performance in initial vegetative growth vigor and larger leaves in hybrids, increasing the synthesis of photosynthesis compounds and enhancing photosynthesis compound synthesis. Comparative transcriptome analysis showed that transgressive down-regulation (TDR) is the main gene expression pattern in the hybrids (*G. hirsutum* × *G. barbadense*, HB), and it was found that the genes of photosystem I and Adenosine triphosphate (ATP)-binding may promote early growth vigor. Transgressive up-regulation (TUR) is the major primary gene expression pattern in the hybrids (*G. barbadense* × *G. hirsutum*, BH), and photosystem II-related genes mediated the performance of early biomass heterosis. The above results demonstrated that overdominance mediates biomass heterosis in interspecific hybrid cotton and the supervisory mechanism divergence of hybrids with different females. Photosynthesis and other metabolic process are jointly involved in controlling early biomass heterosis in interspecific hybrid cotton. The expression pattern data of transcriptome sequencing were supported using the qRT-PCR analysis. Our findings could be useful in theoretical and practical studies of early interspecific biomass heterosis, and the results provide potential resources for the theoretical and applied research on early interspecific biomass heterosis.

## Introduction

Heterosis, also known as hybrid vigor, refers to the phenomenon in which hybrids outperform their inbred parents in terms of development, biomass, yield, and fertility (Hochholdinger and Baldauf, 2018). So far, extensive research has revealed the mechanism of heterosis in rice and maize at various levels (Huang et al., 2016; Xiao et al., 2021) by multi-omics, which dramatically accelerates the use of hybrids in agriculture to meet human needs. During the last century, various hypotheses such as dominance, overdominance, and epistasis (Williams, 1959; Xiao et al., 1995; Yu et al., 1997; Li et al., 2001; Luo et al., 2001; Hua et al., 2003) were proposed to explain the hybrid vigor. In addition, incomplete dominance of alleles was also believed to be the main reason for the hybrid vigor in the preliminary work (Yang et al., 2017). Recently, epigenetics was found to be another molecular mechanism controlling the performance of heterosis through regulating gene expression, and several breakthroughs have been achieved in this field (Dapp et al., 2015; Song et al., 2017; Lauss et al., 2018; Sinha et al., 2020). However, systematically demonstrating heterosis using one of these hypotheses is difficult.

*Gossypium hirsutum* is widely cultivated due to its higher fiber yield and acceptable quality, accounting for more than 90% of the world’s cotton cultivated acreage (National Cotton Council, 2006^1^; Chen et al., 2007; Teodoro et al., 2019), although fiber quality of island cotton is better than that of upland cotton. Cotton breeders have been trying to enhance the yield (Shahzad et al., 2020a) and quality (Li et al., 2018) of upland cotton by utilizing hybrid vigor, and rapid progress is being made in variety breeding. Despite extensive research into fiber yield and quality heterosis, only a few intraspecific upland cotton hybrids have been successfully used in cotton production. Interspecific hybrids were thought to have a lot of potential in crop production because of their hybrid vigors. The main impediment was most likely the hybrids’ massive vegetative growth (Zhang et al., 2014). As a result, interspecific hybrids have received less attention, and only a few have been successfully used in agriculture. Indica-japonica intersubspecific hybrids rice was one of the few intersubspecific hybrids already planted in China. Interspecific hybrids between *G. hirsutum* and *Gossypium barbadense*, meanwhile, received far less attention than intraspecific hybrids, resulting in the discovery of few mechanisms of hybrid vigor.

Biomass heterosis is a widely documented phenomenon at the early seedling development stage of hybrids in multiple species. Previous research has indicated that leaf growth played a pivotal role in biomass heterosis during early vegetative development (Zhu D. et al., 2016), because hybrids own larger leaves, they can provide more photosynthesis area, resulting in more photosynthetic products, such as nutrients, energy, and raw materials for plant growth and development (Liu et al., 2020). *Arabidopsis* hybrids, for example, the process of photosynthesis earlier than their parents during the seedling vegetative development stage, were accompanied by the expression of photosynthesis-related genes such as chloroplast-targeted genes and photosynthetical system genes (Fujimoto et al., 2012). Furthermore, Liu et al. (2021) investigated the transcriptional regulatory network in hybrids together with their parents during early seedling development, and they found that dominant expression complementary capacities between photosynthesis and cell cycle contribute to *Arabidopsis* interspecific heterosis. The two fundamental biological pathways of photosynthesis and auxin biosynthesis play significant roles in biomass heterosis, which were identified in *Brassica napus* hybrids (Zhu et al., 2020). In addition, the balance between primary and secondary metabolism is also likely to serve important function in biomass heterosis in the allodiploid hybrid between *Brassica rapa* and *Raphanus sativus* (Gibum et al., 2020). Similarly, researchers have discovered that genes of the circadian rhythm pathway were down-regulated in leaves and roots in intraspecific hybrids for biomass heterosis of upland cotton (Shahzad et al., 2020b). If mechanisms related to biomass heterosis such as these crucial genes or pathways can be revealed thoroughly after extensive investigation, breeders can probably find a way to inhibit the unnecessary vegetative growth and subsequently enhance the cotton reproductive development to improve the yield and quality.

Genome-wide transcriptome analysis is an efficient method that has been widely used to investigate genes related to biomass heterosis. In this study, we characterized genome-wide transcriptional profiles of reciprocal hybrids (HB and BH) between *G. hirsutum* (GH) and *G. barbadense* (GB) and their parents to explore the function of homologous genes in biomass heterosis during the early vegetative development stage. Genome-wide comparative transcriptome analysis was performed to investigate the molecular bases of regulating biomass heterosis after interspecific hybridization. We presented the dynamic changes of gene expression in hybrids with different females at vegetative development stages to determine the related genes that the polyploid plants express under interspecific hybridization, the processes involved, and how they can be efficiently controlled for plant architecture and rationally solve the problem that vegetative growth is too exuberant to be directly applied in production.

## Materials and Methods

### Plant Materials and Measurement of Early Biomass Heterosis

In a previous study, fiber quality and yield traits of 53 interspecific F_1_ hybrids derived from the crosses between 12 upland cotton and 5 island cotton varieties and their parents were evaluated in Lin’an, Zhejiang and Sanya, Hainan (Li Z. et al., 2020), and the reciprocal F_1_ from crossing between the upland cotton variety Luyuan343 and island cotton variety Achang599 was found having the fiber quality better than their parents. The hybrid HB was developed by the female parent (GH) with the male parent (GB), and its reverse cross BH was developed by crossing the female parent (GB) with the male parent (GH). The seeds of this cross combination were used in this study to investigate biomass heterosis at the seedling stage.

After the cotton seeds were delinted using sulfuric acid, 15 healthy and full seeds were selected from each material. Two uniform seeds of every cultivar were sown in each pot and placed in an artificial climate incubator at 25°C for 16 h light (12000 Lx) and at 20°C for 8 h dark (0 Lx). Eleven days after sowing (DAS), the cotyledons were fully opened, and plant height and leaf area were measured every other day. After 15 DAS, the first true leaf was unrolled and the third true leaf emerged at 21 DAS. We stopped measuring phenotypic traits and biomass heterosis after the appearance of the third true leaf. Six plants of each material were randomly selected to determine the plant height, leaf area, and biomass heterosis estimation. Quantitative analysis of the leaf area of the parents and F_1_ hybrids at different stages using Image-J software as Syed and Khan (2018) described. Fresh biomass of the whole plant was measured and dried in a thermostat oven for 2 h (105°C) and thoroughly dried in a thermostatic blast oven at 80°C.

MS Excel and SPSS (22.0) software were used for statistical analysis, and the significant difference analysis was performed using Student’s *t*-test. Mid-parent heterosis (MPH) and over-parent heterosis (OPH) of the F_1_ hybrid over its parents for biomass were calculated as follows (Sundrish et al., 2010):

MPH = (F1 – mean P)/mean P in percent

OPH = (F1 – best P)/best P in percent

Three cotyledons (cotyledon stage) and the most apical tip of the true leaf (one-leaf stage and trefoil stage) in four materials were taken per sample for the next experiments, frozen in liquid nitrogen after mixing, and stored at –80°C.

### RNA Extraction, Library Construction, Transcriptome Sequencing, and Quantitative Real-Time PCR

The total RNA was extracted from mixed samples using a FastPure^®^ plant total RNA isolation kit (polysaccharides and polyphenolics-rich) (RC401-01, Vazyme Biotech, Co., Ltd., Nanjing, China) according to the manufacturer’s instructions. Two biological replicates were performed for every sample. RNA purity and concentration were determined using a NanoDrop 2000 spectrophotometer (Thermo, Shanghai, China). One microliter of the extracted RNA was subjected to 1% agarose gel electrophoresis to check the degradation and contamination. In total, 24 RNA-seq libraries with a data volume of 6 G comprising four cotton materials (GH, GB, HB, BH) at three developmental stages (cotyledon stage, one-leaf stage, and trefoil stage) were constructed using an NEBNext Ultra™ II RNA Library Prep Kit (NEB, E7775), and 125 bp/150 bp paired-end sequences were generated using an Illumina Hiseq™ 2500 platform.

A reverse transcription kit (R212-01, Vazyme Biotech Co., Ltd.) was used to perform reverse transcription to synthesize cDNAs. The quantitative real-time PCR (qRT-PCR) was carried out using the two-step RT-PCR with CFX Connect Real-Time System (Bio-Rad, Hercules, CA, United States) and analyzed by the 2^–ΔΔCT^ method.

### Data Quality Control and Alignment of RNA-Seq Reads to the Reference Genomes

Before being aligned to the reference genome, adapter-contaminated and low-quality reads were removed from all 24 samples’ raw reads using Cutadapt (Kechin et al., 2017) and FastQC software (FastQC^2^). The clean reads of parent samples (GH and GB) were mapped by HISAT 2 (Kim et al., 2019) against the updated genome sequences of *G. hirsutum* (TM-1_ZJU v2.1) and *G. barbadense* (H7124_ZJU) (Hu et al., 2019). The clean reading of F_1_ hybrids was mapped to the sum of the reference genomes of the parents. The reference genome data were downloaded from COTTONGEN (Yu et al., 2014). The number of reads uniquely mapped to transcribed regions of each gene was extracted for all gene fragments per kilobase per million mapped reads (FPKM), and the reads mapping on multiple positions were discarded (Mortazavi et al., 2008). The differentially expressed genes (DEGs) with | log2 (fold change)| > 1 and an adjusted (*p*-value < 0.05) by DEseq2 (Love et al., 2014) were determined.

### Homologous Gene Alignment Between Two Reference Genomic

Protein sequences between *G. hirsutum* genome and *G. barbadense* genome were aligned by BLAST (Altschul et al., 1997) to identify the corresponding relationship of homologous gene pairs (*E*-value, <10^–5^), which were considered to be homologs having at least 90% sequence in two genomes, and only one pair of homologs depending on the above relationship of orthologous genomic regions was screened as a homologous gene.

### Expression Patterns Analysis, Functional Annotation, and Classification of Overdominant Genes

The expression level dominance (ELD) analysis in F_1_ hybrids compared to their parents was divided into five categories (Figure 3A), total of 12 expression patterns including additivity (I and XII), GH-expression level dominance (ELD-H, IV and IX), GB-expression level dominance (ELD-B, II and XI), transgressive up-regulation (TUR, V, VI, and VIII), and transgressive down-regulation (TDR, III, VII, and X) using STEM (Short Time-series Expression Miner) software as described by Rapp et al. (2009).

The genes annotation from different expression patterns for gene ontology (GO) enrichment analysis using *G. hirsutum* genome annotation was performed by the OmicShare tools, a free online platform for data analysis,^3^ with corrected *p* < 0.05 as the significantly enriched and rich factor.

## Results

### Early Establishment of Growth Vigor in Interspecific Hybrids

**FIGURE 1:**
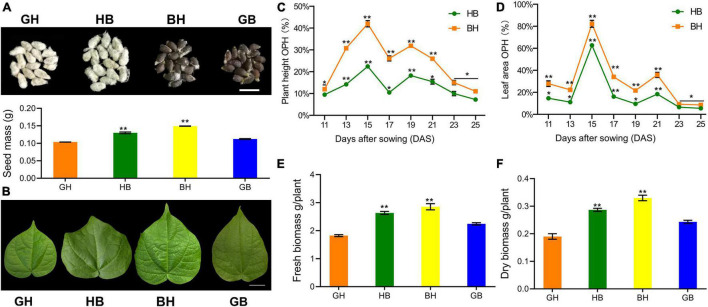
Biomass heterosis exists in interspecific hybrids at the early seedling stage. **(A)** Seed morphology (upper panel) and weight (lower panel, means ± SEM) in parents (GH and GB) and their reciprocal F_1_ hybrids (HB: GH × GB and BH: GB × GH). Scale bar = 10 mm. **(B)** Leaf morphology and area at one-leaf stages in GH, GB, and their hybrids. Scale bar = 10 mm. **(C)** Plant height over-parent heterosis (OPH) performance of HB and BH (mean ± SEM, *n* = 6). **(D)** Leaf area OPH performance of HB and BH (mean ± SEM, *n* = 6). **(E)** Fresh biomass observed in hybrids relative to their parents (mean ± SEM, *n* = 6). **(F)** Dry biomass observed in hybrids relative to their parents (mean ± SEM, *n* = 6). Here ** is used for significant difference with high parents (HP, GB) performance at *p* < 0.01 and * at *p* < 0.05.

It has been reported that the heterosis of seedling growth vigor may be related to the seed mass (East, 1936); however, this hypothesis was not upheld by the research of heterosis in maize inbred lines hybrids (Ko et al., 2016). In addition, our study depicted that the seed mass of reciprocal F_1_ hybrids was significantly higher than that of the parents (Figure 1A), seedling growth vigor heterosis occurred soon after germination, and it was significantly greater than in the high parents at the cotyledons unrolled stage (Supplementary Figure 1) and persisted throughout the seedling stage (Figures 1A,B and Supplementary Figure 1).

The measurement of plant height and leaf area started 11 DAS when the cotyledons were unrolled in parents and F_1_ hybrids. Plant height and leaf area of F_1_ hybrids showed significant or highly significant OPH from the first measurement (Figures 1C,D). Both biomass traits peaked at 15 DAS (one-leaf stage). The dry weight and fresh weight of the one-leaf stage were measured, and it was found that the reciprocal F_1_ hybrids were significantly greater than the HP value (GB, Figures 1E,F).

The leaf is the primary photosynthesis organ in plants and the primary source of storage and utilization of essential substances for growth (Boeven et al., 2020). F_1_ hybrids had greater leaf area and plant height than their parents, which was highly significant at the first true leaf unrolled stage (Figures 1C,D). The OPH of plant height is greater than 40% (Figure 1C), and the OPH of leaf area is more than 80% (Figure 1D). Moreover, there is a lower peak that occurred at the third true leaf unrolled (Leaf area: 21 DAS) or 2 days prior to the trefoil stage (Plant height: 19 DAS). Then, the heterosis decreased from extremely meaningful to a significant level, the trend essentially flattened and stabilized at the OPH of about 10%.

### Homologous Expression Divergence Is Caused by Hybridization During the Early Vegetative Growth Development Stage

We harvested the leaves of three representative stages: cotyledon stage, one-leaf stage, and trefoil stage according to the above results (Figures 1C,D), and transcriptome sequencing was performed subsequently. Leaf tissues of the parents (GH and GB) and hybrids (HB and BH) at three stages were used to build 24 cDNA libraries for sequencing. The overall sequencing means of total mapped was 94.74%, and the value of Q30 was 91.49% in our sequencing data. In the leaf of GH, GB, HB, and BH, the mean of clean reads were approximately 43.62, 45.63, 43.60, and 43.85 million, respectively. In the parents, more than 91.87 and 93.70% mean of unique reads were mapped to the *G. hirsutum* and *G. barbadense* reference genome, respectively. In the F_1_ hybrids, the mean of unique reads was 41.69 and 42.21%, which is due to mapping to the addition of the two sets of reference genomes. Therefore, the mean number of reads mapped to the gene region of parents and hybrids was ∼85.68, 80.31, 79.50, and 86.35%, respectively (Supplementary Table 1).

**FIGURE 2:**
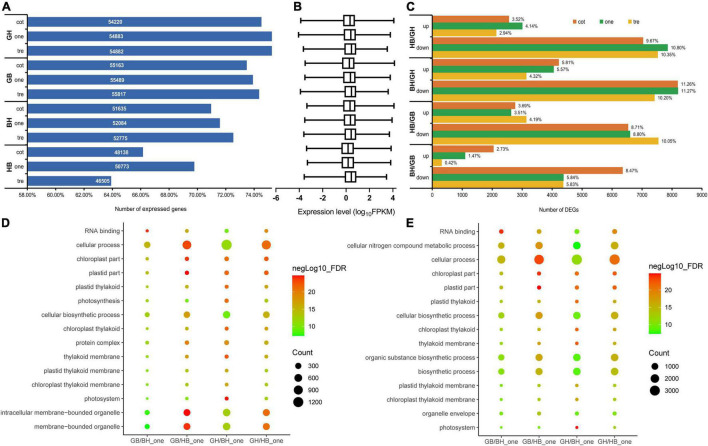
Transcriptome sequencing of the parents (GH and GB) and their reciprocal hybrids (HB and BH). **(A)** The number and percentage of genes expressed in the four materials at three vegetative growth development stages. **(B)** Expression levels in the four materials at three vegetative growth development stages. **(C)** Differential expression genes in F_1_ hybrids with their parents at a different stage. **(D,E)** The top 15 terms from GO enrichment analysis of DEGs at one-leaf stage, **(D)** up-regulation, and **(E)** down-regulation. cot, cotyledon stage; one, one-true leaf stage; tre, trefoil stage.

Analysis of transcript accumulation patterns in parents relative to their reciprocal F_1_ hybrids, an average of genes expressed at different stages was investigated by RNA-seq (Figure 2A). The average expression level of the F_1_ hybrids compared with the parents shows minor differences, but it is not significant (Figure 2B).

**FIGURE 3:**
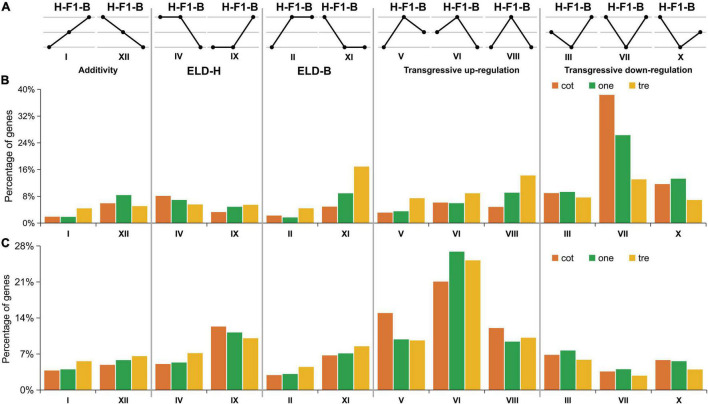
The 12 possible differential expression patterns in F_1_ hybrids compared with their parents at different stages. **(A)** Expression patterns of 12 groups, H: *Gossypium hirsutum*; F_1_: hybrid, and B: *Gossypium barbadense*. Roman numerals indicate the same categorization as used in Rapp et al. (2009). **(B)** The percentage of genes from the 12 patterns in HB relative GH and/or GB. **(C)** The percentage of genes from the 12 patterns in BH relative GH and/or GB. cot, cotyledon stage; one, one-true leaf stage; tre, trefoil stage.

To study the gene expression patterns after interspecific hybridization, we first established the relationship between homologous gene pairs among two reference genomes by whole-genome alignment and aligned the expression profile according to the homologous gene pairs of *G. hirsutum* and *G. barbadense* to construct the interworking best hits.

The proportion of DEGs between hybrids with different females and their parents was different at three seedling stages (Figure 2C). The largest differentially up-regulated genes after hybridization were found in BH when compared with the male parent GH (15.70%), and the largest down-regulated genes were found in BH when compared with GH too (32.73%). The BH with maternal GB had the fewest DEGs that were up-regulated and down-regulated after hybridization, with 4.62 percent and 20.13 percent, respectively (Figure 2C). DEGs analysis of hybrids and parents found that the number of down-regulated genes was significantly higher than the up-regulated genes.

To explore the specific biological processes that were enriched in the process at the one-leaf stage, we classified the DEGs in the top 50 and selected the top 15 according to ascending order of FDR value and rich factor. Assessment of biological processes for the up- and down-regulated DEGs in hybrids and parents found that most genes were enriched in the same GO terms, involved in the chloroplast thylakoid membrane, chloroplast part, chloroplast thylakoid, photosystem, cellular biosynthetic process, cellular process, plastid part, plastid thylakoid, plastid thylakoid membrane, thylakoid membrane, and RNA binding. DEGs involved in photosynthesis, intracellular membrane-bounded organelle, membrane-bounded organelle, and protein complex were upregulated, whereas the DEGs involved in the biosynthetic process, cellular nitrogen compound metabolic process, organelle envelope, organic substance biosynthetic process were downregulated in both the GB vs. BH, GB vs. HB, GH vs. BH, and GH vs. HB comparisons (Figures 2D,E). Nevertheless, a portion of the allele derived from the parent after hybridization were both up- and down-regulated the genes related to the chloroplast, cellular process, and plastids. Hybrids strengthened the nitrogen metabolism and organelles in the remaining items while weakening the membrane binding.

### Genome-Wide Homologous Genes Expression Patterns Divergence of Reciprocal F_1_ Leads to Different Mechanisms of Biomass Heterosis

Expression level dominance has been discovered in allopolyploids, a phenomenon in which progeny gene expression is statistically similar to that of one parent (Shen et al., 2017; Boeven et al., 2020), including asymmetric expression patterns in the polyploidy process from two diploids to tetraploid cotton (Yoo et al., 2012). In this section, we classified the expression patterns of the genes in parents and hybrids into 12 groups and divided them into five categories, including Additive (I and XII), ELD-H (IV and IX), ELD-B (II and XI), TUR (V, VI, and VIII), and TDR (III, VII, and X) according to the description of Rapp (Rapp et al., 2009; Figure 3A).

The results showed the expression pattern of transgressive regulation with the largest number of genes in HB hybrids (Figure 3B), among which the pattern of TDR contained more genes than TUR. In the ELD of parents, it can be observed that the number of genes of ELD-B (13.01%) is more than that of ELD-H (11.41%), but the difference was not significant (less than 2%). Among the five categories of expression patterns, additives contained the least number of genes (9.17%). The patterns in BH showed transgressive regulation contained the largest number of genes (61.79%), the same as HB, in which TUR had the largest gene classification, followed by ELD-H (17.04%), which was different from HB (Figure 3C). And, the third was TDR (15.40%), followed by ELD-B (10.95%) and additives (10.22%).

Taken together, the interspecific heterosis was possibly due to the transgressive regulation, but the reciprocal F_1_ hybrids (HB and BH) exhibited a distinct expression pattern. In F_1_ hybrids (both HB and BH), the expression pattern transgressive regulation (including TUR and TDR) exceeds 60% of the total expressed genes. The biomass heterosis of HB with upland cotton as a female parent was generated through the expression pattern of TDR, while the expression pattern of TUR contributed to the heterosis of BH with island cotton as a female parent. Furthermore, the results of expression pattern analysis revealed that overdominance at the genome expression level plays an unusual role in the early biomass heterosis in interspecific hybrid cotton.

### The Comparison of Overdominance Genes Between Reciprocal Hybrids

**FIGURE 4:**
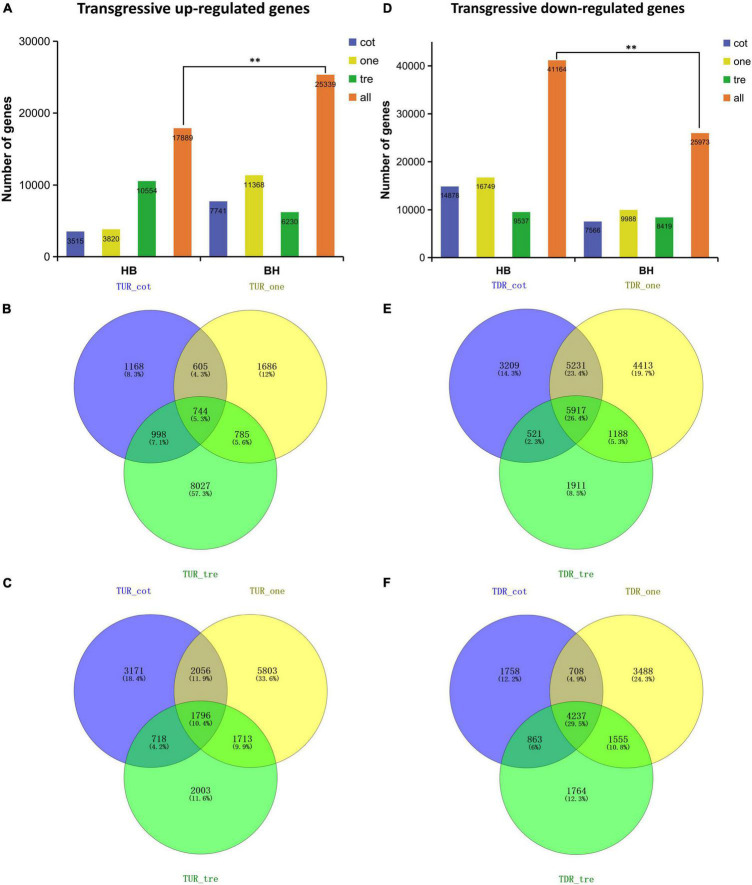
Quantification of genes that showed transgressive regulation patterns from the three different stages. **(A,D)** The number of transgressive up- and down-regulation expression patterns at three different stages. **(B)** Transgressive up-regulation (TUR) expression pattern in HB. **(C)** TUR expression pattern in BH. **(E)** Transgressive down-regulation (TDR) expression pattern in HB. **(F)** TDR expression pattern in BH represents the distribution of unique and common expression genes. Here ** is used for the number of transgressive regulated genes in HB performed significant difference with that in BH at *p* < 0.01.

The expression divergence of overdominance genes (TUR and TDR) at different seedling stages was compared to investigate the gene expression pattern in reciprocal hybrids at early biomass vigor. The findings revealed that the total number of transgressive up-regulated genes in BH was significantly higher than in HB, but not in TDR (Figures 4A,B).

A comparison of the transgressively up-regulated genes expressed in HB at various seedling stages revealed that the number of expressed genes increased over time, but there was no significant difference between the cotyledon stage and the one-leaf stage (Figure 4A). Co-expression analysis of genes at different seedling stages showed that 744 genes were co-expressed at three developmental stages in HB (Figure 4B). Differences among BH and HB in the number of transgressive up-regulated genes were observed from cotyledon stages and peaked at the one-leaf stage, followed by the cotyledon stage and the three-leaf stage (Figure 4A), and 1796 genes were co-expressed at these three stages in BH (Figure 4C). The number of transgressive down-regulated genes expressed at various seedling stages revealed that two hybrids possessed the greatest number of expressed genes at the one-leaf stage (Figure 4D). Co-expression analysis of the TDR genes expressed in HB found that 5917 genes were co-expressed in these three stages (Figure 4E), whereas 4237 genes were co-expressed in BH (Figure 4F).

An overall comparison of TUR and TDR genes expression at different developmental stages and comparative co-expression analysis of genes at all seedling developmental stages suggested a tendency for an increased number of expressed genes at the one-leaf stage, except for TUR genes of HB, which complies with our previous phenotypic analysis (Figure 1). These findings indicated that an increase in the number of expressed genes could have been just one of many factors contributing to biomass heterosis. Meanwhile, differentially expressed patterns were performed in hybrids with different females, and co-expressed genes play important roles at different stages.

### Functional Annotations of Overdominance Genes

To better understand the functions of the overdominant homeolog expression genes in hybrids and parents during early vegetative development, GO enrichment analysis was performed on these genes, which had previously been identified as commonly expressed (Figures 4C,E).

**FIGURE 5:**
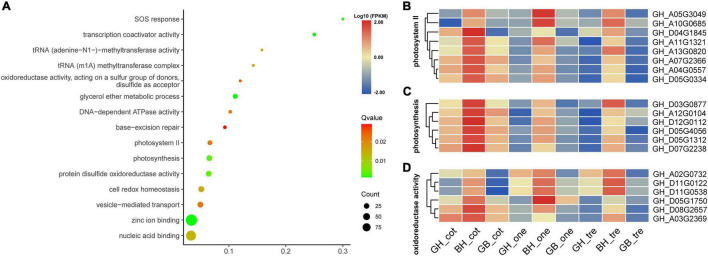
Homolog expression levels in the BH relative to the levels in its parents explain the phenomenon of TDR. **(A)** The significantly enriched GO terms of TDR genes from BH relative to its parents. **(B–D)** The expression heatmap of homologous genes with different stages from enriched GO terms of photosystem II, photosynthesis, and oxidoreductase activity.

Gene ontology enrichment analysis of transgressive up-regulated (TUR) genes in BH compared with its parents was enriched in 26 terms (*p* value < 0.05), such as glycerol ether metabolic process, transcription coactivator activity, photosystem II, photosynthesis, and oxidoreductase activity (Figure 5A). Heatmaps were created to display the expression patterns and clustering of photosystem II, photosynthesis, and oxidoreductase activity genes in BH and its parents (Figures 5B–D). Furthermore, at three different developmental stages, the gene expression representation of BH was both higher and more consistent than that of its parents.

**FIGURE 6:**
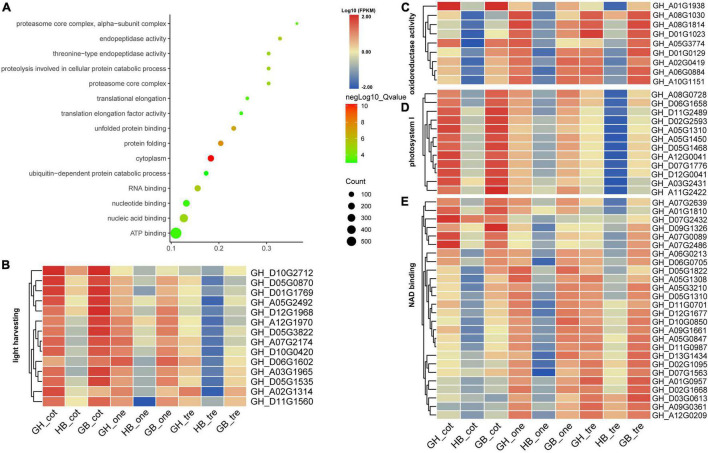
Homolog expression levels in the HB relative to the levels in its parents explain the phenomenon of TUR. **(A)** The significantly enriched GO terms of TUR genes from BH relative to its parents. **(B–E)** The expression heatmap of homologous genes with different stages from enriched GO terms of light-harvesting, oxidoreductase activity, photosystem I, and NAD binding.

Conversely, the transgressive down-regulated (TDR) gene expression pattern in HB compared with its parents found that match 172 Go terms in summary (*p* value < 0.05) compared with its parents and selected the top 15 display with the lowest *p* value (Figure 6A). The most significantly enriched terms were the cytoplasm; following enriched terms such as protein folding, unfolded protein binding, and RNA binding, it was found that Adenosine triphosphate (ATP) binding terms enriched the most DEGs. In addition, we found that the terms related to photosynthesis (light-harvesting, oxidoreductase activity, photosystem I, and NAD binding) were also enriched. Heatmap display of gene expression patterns of TDR is related to photosystem I, oxidoreductase activity, light-harvesting, and NAD binding genes in HB and its parents (Figures 6B–E). These results suggested that the expression of HB in photosynthesis-related genes was lower than that of parents in three stages.

All these results indicate that up- or down-regulation of photosynthesis and ATP-related genes might be the reason for initial biomass heterosis. Furthermore, genes that regulate heterosis in the photosynthesis system were different; the majority of the up-regulated genes were enriched in photosystem II, such as genes containing Psb-related domains. Contrarily, the majority of the down-regulated genes were enriched in photosystem I, photosystem I reaction center, and light-harvesting, such as CAB, LHC family, and genes containing Psa-related domains. This result was consistent with a study on early biomass heterosis in *B. napus (Canola)* hybrids (Zhu et al., 2020). Additionally, down-regulated genes included the NAD binding genes to reduce hydrogen ions to H_2_.

### Expression Pattern Verification by Quantitative Real-Time PCR

**FIGURE 7:**
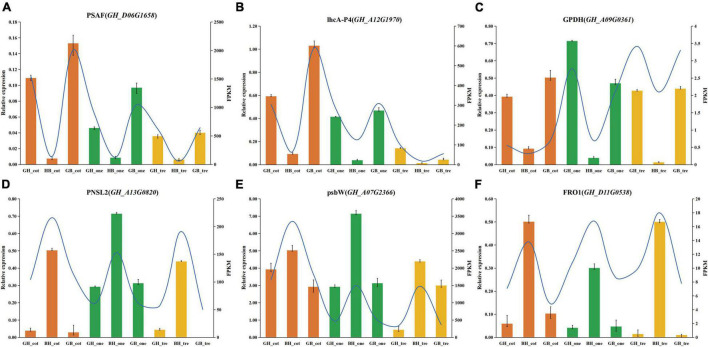
Validation of transcriptional data changes of selected genes from expression patterns of TUR and TDR using the qRT-PCR in hybrids and their parents. **(A–F)** Comparisons of RNA-seq data (FPKM value; blue color lines) with the qRT-PCR (By relative expression data; orange, green, and yellow color columns denote cotyledon stage, one-true leaf stage, and trefoil stage, respectively) of selected genes at three time points during early biomass stages; Panels **(A–C)** indicate genes from HB and panels **(D–F)** indicate genes from BH.

Validation of RNA-Seq expression pattern data with qRT-PCR analysis at three early seedling stages in parents and hybrids was done, and the results matched well with the RNA-Seq analysis described earlier (Figure 7). The qPCR results confirmed the trend in expression of these selected genes including that pathway in the photosystem I (*PSAF*), light-harvesting (*lhcA-P4*), NAD binding (*GPDH*), photosystem II oxygen-evolving complex (*PNSL2*), photosystem II (*psbW*), and oxidoreductase activity (*FRO1*), despite some differences in expression levels. Three genes from HB showed a tendency of down-regulation, whereas three genes from BH, exhibited a trend of up-regulation.

## Discussion

### Overview of Early Biomass Heterosis and Comparative Transcriptome Analysis

Higher fiber yields and quality can be achieved by reducing vegetative growth in favor of reproductive growth, which has been a major goal of cotton breeding. This may be especially important in elucidating the mechanism of early biomass heterosis in order to better serve breeding efforts in that case. It has been found that hybrids exhibited stronger vegetative growth vigor at the seedling stage for essential crops such as rice (Zhu D. et al., 2016), maize (Li Z. et al., 2020), wheat (Liu et al., 2018), and cotton (Ding et al., 2021) in previous research.

In this study, we found that the maximum OPH at the first true leaf (one-leaf stage), followed by decreasing trend that gradually leveled off, similar findings have been reported in maize (Liu et al., 2018). However, there were no significant differences between hybrids and their parents in the contents of chlorophyll per unit area determined (Supplementary Figure 2), the results comply with previous researches on chlorophyll fluorescence parameters determined in *Arabidopsis* (Zhu A. Y. et al., 2016). We inferred that the one-leaf stage is the critical period in early biomass heterosis performance integrated with biomass-related traits determination and the findings of other research, but this phenomenon may not be responsible for the contents of chlorophyll or the intensity of photosynthesis between hybrids and their parents. Certainly, the OPH in the leaf area of reciprocal hybrids compared to their parents causes increased photosynthesis, which exhibits significant interspecific biomass heterosis.

Transcriptome analyses of leaves from reciprocal hybrids and their parents at three seedling stages revealed that the number of expressed genes in hybrids was lower than in parents, particularly in crosses with GB as the female. However, there were no significant differences in gene expression levels between hybrids and parents. This indicates that the performance of biomass heterosis contributed to the inhibition of gene expression after hybridization, similar results have been recorded in maize (Luo et al., 2021), tetraploid *Medicago sativa* (Li et al., 2009), and intraspecific hybridization of upland cotton.

### Expression Patterns Divergence of Reciprocal F_1_ Hybrids After Hybridization and Both Overdominant Genes Mediate Early Biomass

The evolution of cotton from diploid to tetraploid was a known process of natural hybridization and chromosome doubling. Previous research on the phenomenon of subgenome asymmetry and ELD during hybridization and evolution from diploid to tetraploid in cotton has been published (Peng et al., 2020). Of course, this pattern is largely determined by the up- or down-regulation of homologous genes in non-dominant parents. Furthermore, Yoo et al. (2012) found that new gene expression conditions are selected for evaluation by adjusting the mismatches in natural diploid genomic mergers. Cotton interspecific hybrids exhibited heterosis for yield and quality traits. Breeders struggle to achieve considerable heterosis in intraspecific upland cotton crosses, but it is an intricated task. Early-stage biomass production influences plant yield increases. Keeping this in mind, we used comparative transcriptome analysis to evaluate the genetic basis of early biomass heterosis in hybrid cotton of reciprocal crosses.

**FIGURE 8:**
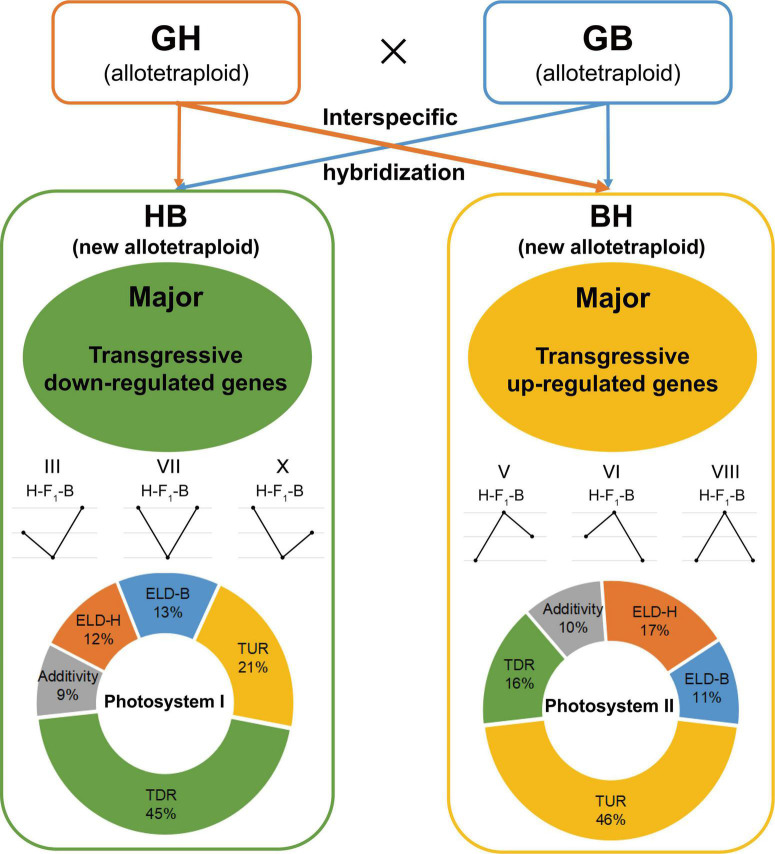
Graphic illustration of early biomass vigor over-parent heterosis (OPH) performance by overdomiant regulated genes in interspecific hybrids and their parents. GH, GB, HB, and BH are the abbreviation of *G. hirsutum*, *G. barbadense*, *G. hirsutum* × *G. barbadense*, and *G. barbadense* × *G. hirsutum*, respectively. The circle proportion refers to the average proportion of different five types [expression level dominance-GH (ELD-H), expression level dominance-GB (ELD-B), transgressive up-regulation (TUR), transgressive down-regulation (TDR), additivity] in three stages. The representation of III, VII, and X (belonging to TDR) and V, VI, and VIII (belonging to TUR) is consistent with that of Figure 3.

Previous studies of comparative transcriptomics analysis in root and leaf found that overdominant gene expression is the major pattern of early biomass heterosis (Shahzad et al., 2020b). These findings comply with the overdominance hypothesis as overdominance was found to be activated in gene expression for upland cotton intraspecific hybrids at different development stages. However, interspecific hybridization has experienced gene flow among different subspecies; whether the hybridization between interspecific and intraspecific represent the exact underlying mechanism remains unknown. It has been reported that overdominant loci are the key genetic factors of lint yield heterosis in interspecific hybrids between *G. hirsutum* and *G. barbadense* (Tian et al., 2019); similarly, Zhao et al. (2009) investigated the mechanism of cotton heterosis in four different stages of growth and development. They detected quantitative and qualitative gene expression differences between hybrids and parents and hypothesized that overdominant gene expressions influenced heterosis (Zhao et al., 2009). The influence of early biomass heterosis was established in our study by differentiating gene expression patterns, inferring the expression of a few genes as additives, similar to that of the male/female parent (ELD) or transgressive up-/down-regulated found by genome-wide expression patterns classification of reciprocal F_1_ hybrids with their parents. Overdominance is the main pattern that regulates the performance of interspecific biomass heterosis, and more than 60% of the genes followed the pattern of transgressive regulation (TUR and TDR). Although the overdominance regulates the performance of interspecific biomass heterosis in both reciprocal F_1_ hybrids, the predominance of up-regulation or down-regulation is opposite in hybrids with a different female parent, and related studies have not been reported previously. We found that TDR is the major pattern in HB (female, *G. hirsutum*), and this is consistent with the previous research on intraspecific hybridization of upland cotton. The hybrids BH with island cotton as a female parent are just the opposite, which is regulated by TUR. Moreover, the expression pattern of these two hybrids shows a trend for the ELD of the paternal genome, this trend was not significantly different (Figure 8, proportion of ELD-male expression in the torus diagram).

### Photosynthesis-Related Genes Play a Critical Role in Early Biomass Interspecific Heterosis

Biomass heterosis is mainly produced by TUR/TDR regulation patterns in reciprocal hybrids (Figure 4). It was found that the differential expression of photosynthesis-related genes plays a key role in interspecific heterosis by the further functional analyses of overdominant genes. This is probably attributed to the higher leaf area, rather than the difference in chlorophyll content or other photosynthetic parameters (Figure 1). The results are shown in *Arabidopsis* interspecific hybrids between *Col*-*0* and *Per*-*1* as well, and it was found that the transcriptional level of hybrids was greater than their parents, even though the internal structure of the leaf is stable (Liu et al., 2021). Likewise, research into the hybrids between *Col* and *C24* found that mature embryo organ sizes or cell numbers were not altered in a leaf unit area (Meyer et al., 2012). These findings are consistent with our previous studies (Li T. Y. et al., 2020).

Functional analysis of transgressive regulation genes found that it was not uniform in expressed genes enriched in photosynthesis between HB and BH. These items were similar to those described above, except that they are both enriched in photosynthesis and oxidoreductase activity terms. It indicated that hybrids are similar in regulating biomass through photosynthesis-related genes, but there are still some differences that TUR genes are mainly enriched in photosystem I, additionally, there are NAD, light-harvesting, and ATP-binding related genes. In contrast, TDR genes were mainly enriched in photosystem II, as well as in terms of cell redox homeostasis and DNA-dependent ATPase activity. Neither biomass nor yield was found to be associated with photosystem II at heterosis in the previous work, which exemplified our hypothesis too. On the other hand, we did not identify any additional relevant studies that suggest that photosystem I is able to maintain a complementary relationship with photosystem II, nonetheless, the repair system in photosystem II is faster than in photosystem I. This might be due to the photosystem I binding chlorophyll *a* and the photosystem II binding chlorophyll *a* and *b* (Chen et al., 2021). It has been found that improving the ability of the light system in maize can promote the heterosis of biomass (Meena et al., 2021). These findings provide a creditable theoretical basis for the high expression of photosystem II related genes to promote the accumulation of photosynthetic products when photosystem I related genes are inhibited.

## Conclusion

It is worth noting that cotton hybrids demonstrated biomass vigor throughout the early phases of vegetative growth. Herein, the one-leaf stage is the most significant stage in early biomass heterosis revealed by comparison of hybrids and their parents, and furthermore, it has indicated that larger leaves in hybrids enhanced photosynthesis compound synthesis. As shown in Figure 8, comparative transcriptome analysis and expression patterns of reciprocal F_1_ hybrids and their parents reveal overdominance mediates interspecific biomass heterosis. However, expression patterns divergence in hybrids with a different female. TDR is the main patterning among the hybrids with upland cotton as a female parent (HB), and it was found that genes of photosystem I and ATP-binding may promote early growth vigor compared to hybrids and their parents. TUR is the major pattern among the hybrids with island cotton as a female parent (BH), and up-regulation of photosystem II-related genes mediated the performance of early biomass heterosis. In parallel, the interaction of the functional gene to exhibit heterosis between photosystem I and photosystem II still needs to be clarified. Our findings add to the body of knowledge in the field of interspecific biomass heterosis between *G. hirsutum* and *G. barbadense*, specifically the mechanism of OPH among hybrids with different female parents. Our findings and hypotheses have a greater potential for application to interspecific hybrid cotton breeding.

## Data Availability Statement

The data presented in the study are deposited in the NCBI repository, BioProject accession number: PRJNA851519.

## Author Contributions

TL: conceptualization, data curation, software, and writing – original draft, review and editing. FW: data curation and software. MY: writing – original draft, review and editing. KLi: software. YQ and HZ: conceptualization. JZ: formal analysis. KLuo and SZ: writing – original draft. YJ: funding acquisition. YZ: writing – original draft, review and editing, and funding acquisition. JR: conceptualization, data curation, writing – review and editing, and funding acquisition. All authors assisted in the critical follow-up of the work and read and approved the manuscript.

## Conflict of Interest

The authors declare that the research was conducted in the absence of any commercial or financial relationships that could be construed as a potential conflict of interest.

## Publisher’s Note

All claims expressed in this article are solely those of the authors and do not necessarily represent those of their affiliated organizations, or those of the publisher, the editors and the reviewers. Any product that may be evaluated in this article, or claim that may be made by its manufacturer, is not guaranteed or endorsed by the publisher.

## Footnotes

1
http://www.cotton.org

2
http://www.bioinformatics.babraham.ac.uk/projects/fastqc/

3
http://www.omicshare.com/tools
