# Supplementary material for: Expression Patterns Divergence of Reciprocal F1 Hybrids Between Gossypium hirsutum and Gossypium barbadense Reveals Overdominance Mediating Interspecific Biomass Heterosis
Source: Front Plant Sci. 2022 Jul 1;13:892805. doi: 10.3389/fpls.2022.892805 (PMC9284264; doi:10.3389/fpls.2022.892805)
Supplement: Supplementary file 1 [file Data_Sheet_1.docx]

**SUPPORTING INFORMATION**

**Expression patterns divergence of reciprocal F_1_ hybrids between *Gossypium hirsutum* and *G. barbadense* reveals overdominance mediating interspecific biomass heterosis.**

**Tengyu Li^a,b,c^, Fuqiu Wang^c^, Muhammad Yasir^a^, Kui Li^d^, Yuan Qin^c^, Jing Zheng^a^, Kun Luo^a^, Shouhong Zhu^b^, Hua Zhang^a^, Yurong Jiang^a^, Yongshan Zhang^b*^, Junkang Rong^a*^**

^a^ Zhejiang Agricultural and Forestry University, Hangzhou 311300, China.

^b^ State Key Laboratory of Cotton Biology, Institute of Cotton Research, Chinese Academy of Agricultural Science, Anyang 455000, China.

^c^ National Key Laboratory of Crop Genetic Improvement, Huazhong Agricultural University, Wuhan 430070, China.

^d^ Institute of Food and Nutrition Development, Ministry of Agriculture and Rural Affairs, Chinese Academy of Agricultural Science, Beijing 100081, China.

* Corresponding authors.

E-mail addresses: tengyuli18@163.com(T. Li), 13938698299@163.com (Y. Zhang), junkangrong@126.com (J. Rong).

**This file including:**

Figure S1

Figure S2

Table S1

**Figure S1. Growth heterosis starts at the early seedling stage**


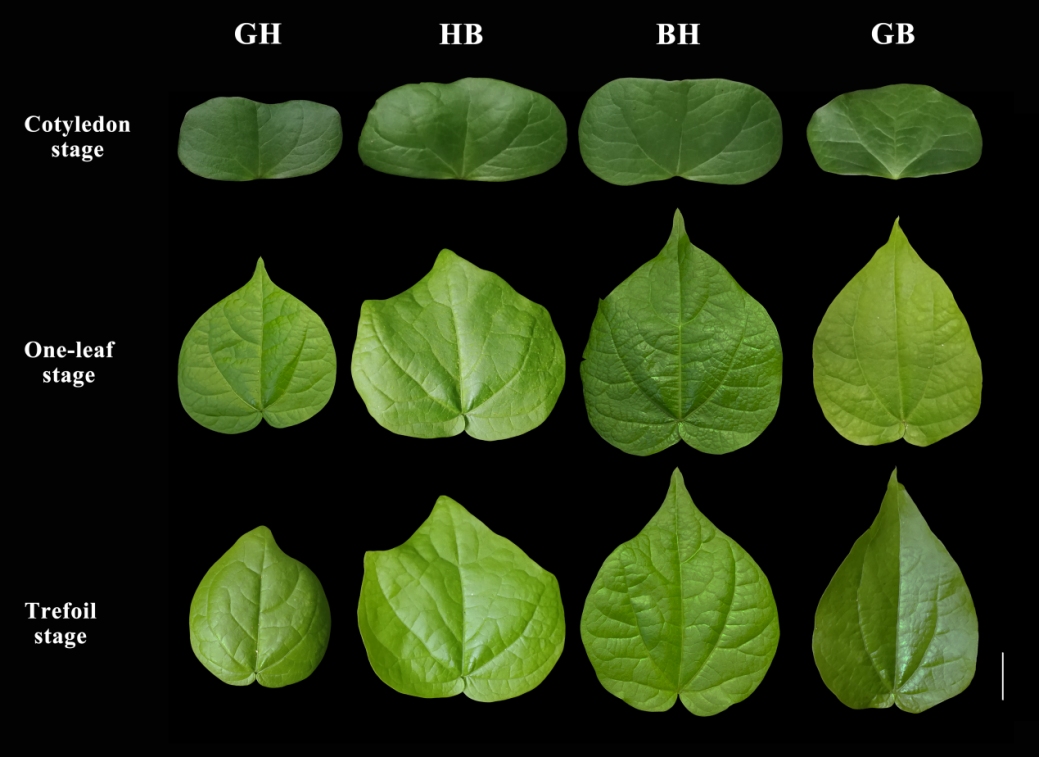


GH, *Gossypium hirsutum*;

HB, *G. hirsutum×G. barbadense*;

BH, *G. barbadense×G. hirsutum*;

GB, *G. barbadense*

**Figure S2. Chlorophyll content at the early seedling stage**

**
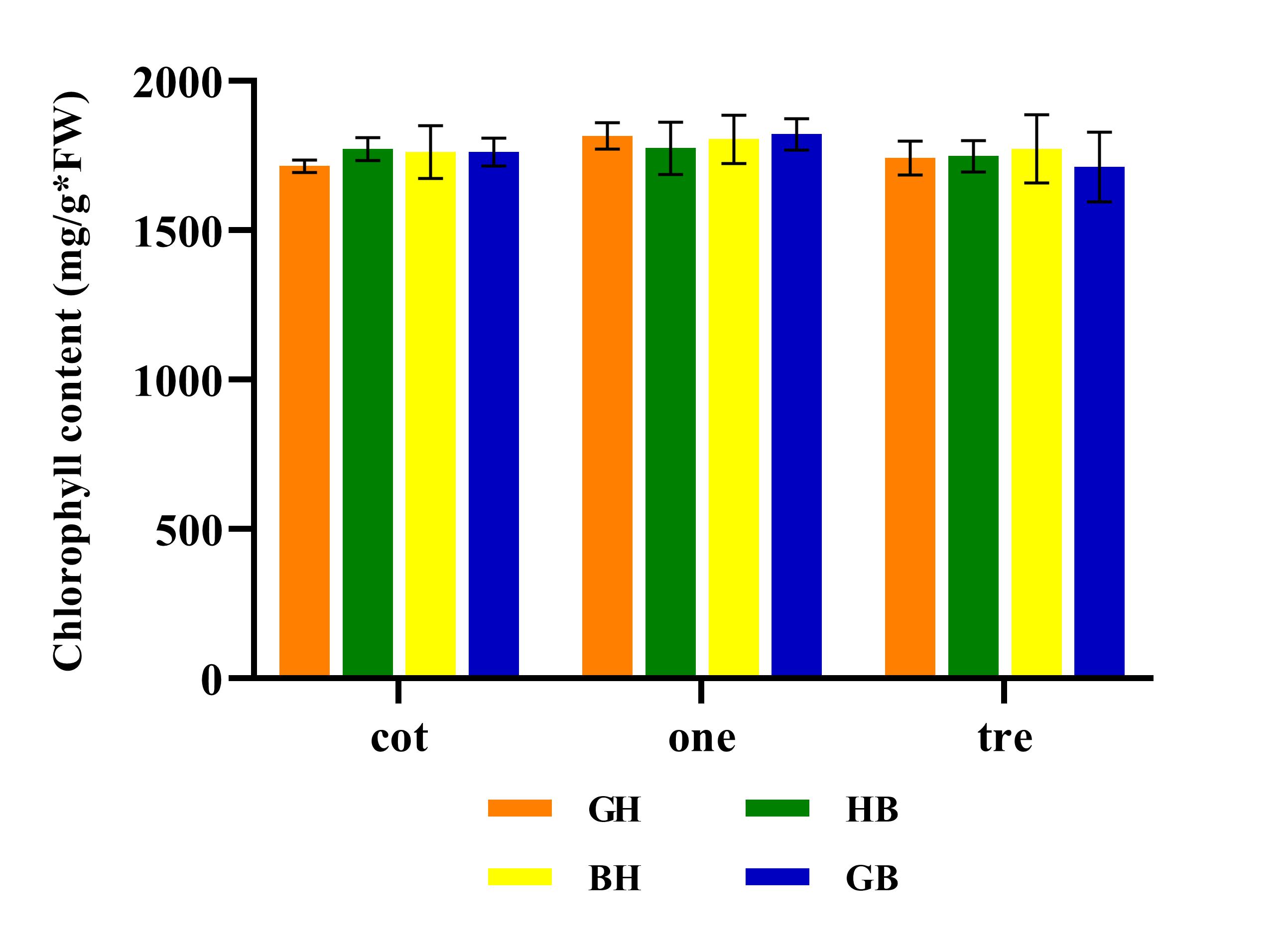
**

**Table S1 Summary of RNA seq data from parents (GH and GB) and hybrids (HB and BH) at different stage**

| Sample name | Raw reads | Clean reads | Total mapped | Multiply mapped | Uniquely mapped | Mapped to Gene | Q30 (%) |
| --- | --- | --- | --- | --- | --- | --- | --- |
| GH_cot_1 | 48.89 | 45.07 | 43.43(96.36%) | 3.94(9.07%) | 39.49(90.93%) | 34.25(86.73%) | 90.53 |
| GH_cot_2 | 47.59 | 43.91 | 42.38(96.51%) | 3.60(8.50%) | 38.78(91.50%) | 33.81(87.19%) | 91.29 |
| GH_one_1 | 49.57 | 45.84 | 44.22(96.47%) | 3.40(7.70%) | 40.81(92.30%) | 34.94(85.62%) | 91.12 |
| GH_one_2 | 45.65 | 42.16 | 40.69(96.53%) | 3.06(7.52%) | 37.64(92.48%) | 32.23(85.63%) | 91.08 |
| GH_tre_1 | 49.15 | 45.40 | 43.82(96.51%) | 3.85(8.78%) | 39.97(91.22%) | 33.72(84.36%) | 91.33 |
| GH_tre_2 | 42.55 | 39.35 | 37.89(96.29%) | 2.74(7.24%) | 35.15(92.76%) | 29.70(84.52%) | 90.90 |
| HB_cot_1 | 47.04 | 43.54 | 41.09(94.38%) | 24.32(59.19%) | 16.77(40.81%) | 13.61(81.12%) | 90.73 |
| HB_cot_2 | 44.69 | 41.27 | 39.12(94.79%) | 23.35(59.69%) | 15.77(40.31%) | 12.80(81.18%) | 91.19 |
| HB_one_1 | 47.82 | 44.25 | 41.36(93.47%) | 24.62(59.53%) | 16.74(40.47%) | 13.49(80.56%) | 91.00 |
| HB_one_2 | 49.09 | 45.37 | 42.73(94.19%) | 24.86(58.19%) | 17.87(41.81%) | 14.38(80.50%) | 90.56 |
| HB_tre_1 | 48.94 | 45.26 | 42.48(93.86%) | 24.02(56.53%) | 18.47(43.47%) | 14.56(78.83%) | 90.86 |
| HB_tre_2 | 45.28 | 41.91 | 39.28(93.72%) | 22.27(56.71%) | 17.00(43.29%) | 13.55(79.67%) | 91.05 |
| BH_cot_1 | 51.01 | 47.25 | 44.70(94.61%) | 26.30(58.85%) | 18.39(41.15%) | 14.88(80.88%) | 90.79 |
| BH_cot_2 | 49.72 | 46.05 | 43.46(94.38%) | 25.50(58.67%) | 17.96(41.33%) | 14.53(80.89%) | 90.63 |
| BH_one_1 | 46.58 | 43.12 | 40.57(94.08%) | 23.51(57.94%) | 17.06(42.06%) | 13.66(80.07%) | 90.57 |
| BH_one_2 | 45.47 | 42.08 | 39.63(94.16%) | 22.96(57.95%) | 16.66(42.05%) | 13.41(80.46%) | 90.74 |
| BH_tre_1 | 45.17 | 41.83 | 38.91(93.04%) | 21.89(56.24%) | 17.03(43.76%) | 13.16(77.25%) | 90.73 |
| BH_tre_2 | 46.31 | 42.78 | 39.77(92.95%) | 22.70(57.09%) | 17.07(42.91%) | 13.21(77.42%) | 90.83 |
| GB_cot_1 | 45.03 | 41.71 | 39.18(93.92%) | 2.78(7.09%) | 36.40(92.91%) | 31.75(87.23%) | 90.65 |
| GB_cot_2 | 55.62 | 51.97 | 49.33(94.92%) | 3.48(7.06%) | 45.84(92.94%) | 40.01(87.27%) | 92.80 |
| GB_one_1 | 50.61 | 47.10 | 44.90(95.33%) | 2.54(5.65%) | 42.37(94.35%) | 36.68(86.58%) | 94.24 |
| GB_one_2 | 50.34 | 46.91 | 44.47(94.80%) | 2.71(6.09%) | 41.76(93.91%) | 36.01(86.23%) | 94.07 |
| GB_tre_1 | 43.53 | 40.50 | 37.96(93.74%) | 2.37(6.25%) | 35.59(93.75%) | 30.39(85.39%) | 94.03 |
| GB_tre_2 | 49.05 | 45.63 | 43.20(94.68%) | 2.45(5.67%) | 40.75(94.33%) | 34.79(85.37%) | 93.92 |

Sample name: cot: cotyledon stage, one: one-leaf stage, tre: trefoil stage; the -1 and -2 represent two biological replicates.

Raw reads: raw sequencing data.

Clean reads: filtered sequencing data. Percentage: compared to raw data.

Total mapped: sequences that can be mapped to the genome. Percentage: compared to clean data.

Multiply mapped: sequences with multiple alignment positions on the reference genome. Percentage: compared to clean data.

Uniquely mapped: sequences with unique alignment positions on the reference genome. Percentage: compared to clean data.

Mapped to Gene: Mapped the total number of reads to the gene region. The unit of the data in columns 2-7 is millions.

Q30: The percentage of bases with a Phred value greater than 30 to the total bases.
